# (μ-Pyridine-2-carbaldehyde azine)bis­[bis­(triphenyl­phosphine-κ*P*)copper(I)] bis­(tetra­fluorido­borate) dichloro­methane disolvate

**DOI:** 10.1107/S160053680902995X

**Published:** 2009-07-31

**Authors:** Li Yang, Yu Xie, Jianping Zou, Jie Jia, Xiaowei Hong

**Affiliations:** a Key Laboratory of Nondestructive Testing (Ministry of Education), Nanchang Hangkong University, Nanchang 330063, People’s Republic of China; b Key Laboratory of Photochemical Conversion and Optoelectronic Materials, TIPC, CAS, Beijing 100190, People’s Republic of China

## Abstract

In the centrosymmetric title complex, [Cu_2_(C_12_H_10_N_4_)(C_18_H_15_P)_4_](BF_4_)_2_·2CH_2_Cl_2_, the Cu^I^ atom adopts a distorted tetra­hedral geometry, defined by two P atoms from two triphenyl­phosphine ligands and two N atoms from a pyridine-2-carbaldehyde azine ligand. The two Cu atoms are bridged by the centrosymmetric pyridine-2-carbaldehyde azine ligand. The F atoms of the tetra­fluorido­borate anion are disordered over two sites [occupancy factors = 0.68 (5) and 0.32 (5)]. The dichloro­methane solvent mol­ecule is disordered over four sites, with occupancy factors of 0.513 (4), 0.173 (5), 0.141 (5) and 0.173 (5).

## Related literature

For general background to the use of neutral pyridine-azines in the construction of di-, tri- and polynuclear complexes, see: Tuna *et al.* (2003 ▶); Guo *et al.* (2002 ▶); Hamblin *et al.* (2002 ▶). For related structures, see: Mo *et al.* (2006 ▶); Zhou *et al.* (2006 ▶).
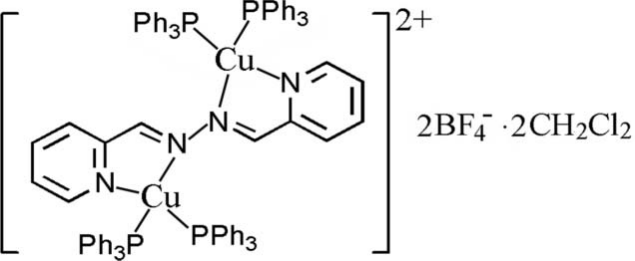


## Experimental

### 

#### Crystal data

[Cu_2_(C_12_H_10_N_4_)(C_18_H_15_P)_4_](BF_4_)_2_·2CH_2_Cl_2_
*M* *_r_* = 1729.89
Monoclinic, 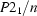

*a* = 13.0932 (16) Å
*b* = 27.501 (3) Å
*c* = 13.9033 (18) Å
β = 115.117 (2)°
*V* = 4532.9 (9) Å^3^
*Z* = 2
Mo *K*α radiation
μ = 0.72 mm^−1^
*T* = 293 K
0.35 × 0.32 × 0.30 mm

#### Data collection

Bruker APEXII CCD diffractometer
Absorption correction: multi-scan (*SADABS*; Sheldrick, 1996 ▶) *T*_min_ = 0.787, *T*_max_ = 0.814
24119 measured reflections
7643 independent reflections
3919 reflections with *I* > 2σ(*I*)
*R*_int_ = 0.063

#### Refinement

*R*[*F* ^2^ > 2σ(*F* ^2^)] = 0.081
*wR*(*F* ^2^) = 0.268
*S* = 0.95
7643 reflections
564 parameters
2 restraints
H-atom parameters constrained
Δρ_max_ = 1.44 e Å^−3^
Δρ_min_ = −0.62 e Å^−3^

### 

Data collection: *APEX2* (Bruker, 2007 ▶); cell refinement: *SAINT* (Bruker, 2007 ▶); data reduction: *SAINT*; program(s) used to solve structure: *SHELXS97* (Sheldrick, 2008 ▶); program(s) used to refine structure: *SHELXL97* (Sheldrick, 2008 ▶); molecular graphics: *SHELXTL* (Sheldrick, 2008 ▶); software used to prepare material for publication: *SHELXTL*.

## Supplementary Material

Crystal structure: contains datablocks global, I. DOI: 10.1107/S160053680902995X/hy2211sup1.cif

Structure factors: contains datablocks I. DOI: 10.1107/S160053680902995X/hy2211Isup2.hkl

Additional supplementary materials:  crystallographic information; 3D view; checkCIF report

## Figures and Tables

**Table 1:** Selected bond lengths (Å)

Cu1—N1	2.061 (6)
Cu1—N2	2.175 (5)
Cu1—P1	2.2741 (19)
Cu1—P2	2.292 (2)

**Fig. 1.**
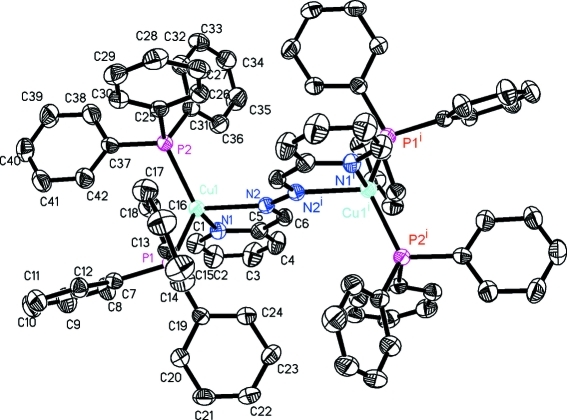
The molecular structure of the title compound. Displacement ellipsoids are drawn at the 30% probability level. H atoms, tetrafluoroborate anion and dichloromethane solvent molecule are omitted for clarity. [Symmetry code: (i) 1 - x, 1 - y, 1 - z.]

## supplementary crystallographic information

### Comment

Neutral pyridine-azines are excellent bridging ligands in coordination chemistry. They are often used to construct some di-, tri- and polynuclear complexes (Tuna *et al.*, 2003; Guo *et al.*, 2002; Hamblin *et al.*, 2002; Zhou *et al.*, 2006). We describe here the synthesis and structure of a new copper(I) compound with a pyridine-2-carbaldehyde azine ligand.

The molecular structure of the title compound is depicted in Fig. 1. The complex is a centrosymmetric dimer with two Cu^I^ atoms bridged by a pyridine-2-carbaldehyde azine ligand. The Cu^I^ atom has a distorted tetrahedral geometry with two P atoms from two triphenylphosphine ligands and two N atoms from the bridging pyridine-2-carbaldehyde azine ligand. The bond angles around the Cu atom are in the range of 77.5 (2)° (N1—Cu1—N2) to 124.02 (7) (P1—Cu1—P2)°. The Cu—P [2.2741 (19) and 2.292 (2) Å] and Cu—N [2.061 (6) and 2.175 (5) Å] bond distances (Table 1) are within the normal ranges for analogous complexes (Mo *et al.*, 2006; Zhou *et al.*, 2006).

### Experimental

Pyridine-2-carbaldehyde and CuBF_4_.4CH_3_CN were prepared by literature method (Mo *et al.*, 2006; Zhou *et al.*, 2006). The title compound was prepared by reacting pyridine-2-carbaldehyde (0.021 g, 0.1 mmol), CuBF_4_.4CH_3_CN (0.031 g, 0.1 mmol) and triphenylphosphine (0.052 g, 0.2 mmol) in 30 ml dry dichloromethane under N_2_ atmosphere. Brown needle crystals suitable for X-ray analysis were obtained by vapor diffusion of diethyl ether into the solution of the title compound in dichloromethane (yield 83%).

### Refinement

H atoms were positioned geomertrically and treated as riding, with C—H = 0.93 (aromatic) and 0.97 (dichloromethane) Å and with *U*_iso_(H) = 1.2*U*_eq_(C). The tetrafluoroborate anion and dichloromethane solvent molecule are disordered. The tetrafluoroborate anion is splitted into two components with site occupancy factor (SOF) values of 0.68 (5) and 0.32 (5). The dichloromethane molecule is splitted into four components with SOF values of 0.513 (4), 0.173 (5), 0.141 (5) and 0.173 (5), respectively. The disorder of the anion and solvent molecule may cause high value of the weighted *R* factor for this structure. The highest peak and deepest hole in the final difference map were associated with atom C45 (at distances of 0.78 Å).

### Crystal data

**Table:**

[Cu_2_(C_12_H_10_N_4_)(C_18_H_15_P)_4_](BF_4_)_2_·2CH_2_Cl_2_	*F*(000) = 1772
*M**_r_* = 1729.89	*D*_x_ = 1.267 Mg m^−^^3^
Monoclinic, *P*2_1_/*n*	Mo *K*α radiation, λ = 0.71073 Å
Hall symbol: -P 2yn	Cell parameters from 24119 reflections
*a* = 13.0932 (16) Å	θ = 2.2–25.0°
*b* = 27.501 (3) Å	µ = 0.72 mm^−^^1^
*c* = 13.9033 (18) Å	*T* = 293 K
β = 115.117 (2)°	Block, brown
*V* = 4532.9 (9) Å^3^	0.35 × 0.32 × 0.30 mm
*Z* = 2	

### Data collection

**Table:**

Bruker APEXII CCD diffractometer	7643 independent reflections
Radiation source: fine-focus sealed tube	3919 reflections with *I* > 2σ(*I*)
graphite	*R*_int_ = 0.063
φ and ω scans	θ_max_ = 25.0°, θ_min_ = 1.5°
Absorption correction: multi-scan (*SADABS*; Sheldrick, 1996)	*h* = 0→15
*T*_min_ = 0.787, *T*_max_ = 0.814	*k* = 0→32
24119 measured reflections	*l* = −16→14

### Refinement

**Table:**

Refinement on *F*^2^	Primary atom site location: structure-invariant direct methods
Least-squares matrix: full	Secondary atom site location: difference Fourier map
*R*[*F*^2^ > 2σ(*F*^2^)] = 0.081	Hydrogen site location: inferred from neighbouring sites
*wR*(*F*^2^) = 0.268	H-atom parameters constrained
*S* = 0.95	*w* = 1/[σ^2^(*F*_o_^2^) + (0.1662*P*)^2^] where *P* = (*F*_o_^2^ + 2*F*_c_^2^)/3
7643 reflections	(Δ/σ)_max_ = 0.001
564 parameters	Δρ_max_ = 1.44 e Å^−^^3^
2 restraints	Δρ_min_ = −0.62 e Å^−^^3^

### Fractional atomic coordinates and isotropic or equivalent isotropic displacement parameters (Å^2^)

**Table:**

	*x*	*y*	*z*	*U*_iso_*/*U*_eq_	Occ. (<1)
Cu1	0.39337 (7)	0.56453 (3)	0.34029 (6)	0.0439 (3)	
F1	0.9059 (12)	0.6834 (11)	0.7796 (13)	0.130 (8)	0.68 (5)
F2	0.919 (3)	0.7544 (5)	0.7121 (13)	0.151 (10)	0.68 (5)
F3	0.956 (3)	0.6852 (17)	0.647 (4)	0.152 (10)	0.68 (5)
F4	1.077 (2)	0.7109 (11)	0.806 (2)	0.152 (8)	0.68 (5)
F1'	1.011 (6)	0.7535 (15)	0.713 (3)	0.17 (2)	0.32 (5)
F2'	1.065 (5)	0.684 (2)	0.799 (5)	0.145 (16)	0.32 (5)
F3'	0.906 (3)	0.721 (2)	0.789 (2)	0.129 (17)	0.32 (5)
F4'	0.910 (5)	0.685 (3)	0.645 (8)	0.123 (15)	0.32 (5)
N1	0.4999 (5)	0.6178 (2)	0.4340 (4)	0.0469 (14)	
N2	0.4973 (5)	0.52483 (19)	0.4841 (4)	0.0437 (13)	
Cl1	0.7434 (6)	0.1422 (2)	0.1283 (7)	0.140 (3)	0.513 (4)
Cl2	0.6074 (8)	0.2242 (3)	0.1389 (9)	0.163 (3)	0.513 (4)
Cl3	0.4482 (17)	0.2880 (6)	0.2405 (19)	0.140 (3)	0.173 (5)
Cl4	0.303 (3)	0.2584 (14)	0.038 (4)	0.163 (3)	0.173 (5)
Cl5	0.687 (2)	0.2339 (8)	0.229 (3)	0.140 (3)	0.141 (5)
Cl6	0.846 (3)	0.2284 (12)	0.451 (3)	0.163 (3)	0.141 (5)
Cl7	0.2701 (18)	0.2418 (7)	0.105 (2)	0.140 (3)	0.173 (5)
Cl8	0.473 (2)	0.2573 (9)	0.090 (2)	0.163 (3)	0.173 (5)
P1	0.44194 (15)	0.54008 (7)	0.20904 (13)	0.0438 (5)	
P2	0.21113 (15)	0.57584 (7)	0.31766 (14)	0.0456 (5)	
B1	0.9692 (15)	0.7102 (6)	0.7357 (12)	0.103 (4)	
C1	0.5039 (7)	0.6647 (3)	0.4100 (7)	0.064 (2)	
H1	0.4538	0.6759	0.3435	0.077*	
C2	0.5824 (8)	0.6983 (3)	0.4827 (8)	0.082 (3)	
H2	0.5860	0.7302	0.4621	0.098*	
C3	0.6508 (8)	0.6832 (3)	0.5813 (8)	0.088 (3)	
H3	0.6985	0.7050	0.6314	0.106*	
C4	0.6489 (7)	0.6349 (3)	0.6066 (7)	0.074 (2)	
H4	0.6985	0.6235	0.6731	0.089*	
C5	0.5730 (6)	0.6032 (3)	0.5329 (6)	0.0514 (18)	
C6	0.5683 (6)	0.5522 (2)	0.5556 (5)	0.0482 (17)	
H6	0.6163	0.5395	0.6213	0.058*	
C7	0.3910 (6)	0.5791 (3)	0.0920 (5)	0.0484 (17)	
C8	0.4119 (8)	0.6284 (3)	0.1071 (6)	0.067 (2)	
H8	0.4525	0.6402	0.1756	0.080*	
C9	0.3739 (8)	0.6607 (3)	0.0231 (7)	0.081 (3)	
H9	0.3866	0.6939	0.0347	0.097*	
C10	0.3171 (8)	0.6428 (3)	−0.0782 (7)	0.075 (2)	
H10	0.2902	0.6640	−0.1356	0.090*	
C11	0.3000 (7)	0.5942 (3)	−0.0948 (6)	0.067 (2)	
H11	0.2636	0.5824	−0.1638	0.080*	
C12	0.3363 (6)	0.5620 (3)	−0.0101 (5)	0.0536 (18)	
H12	0.3237	0.5289	−0.0224	0.064*	
C13	0.3871 (6)	0.4803 (2)	0.1559 (5)	0.0468 (17)	
C14	0.4509 (7)	0.4451 (3)	0.1386 (7)	0.065 (2)	
H14	0.5260	0.4513	0.1535	0.079*	
C15	0.4029 (9)	0.3994 (3)	0.0981 (8)	0.085 (3)	
H15	0.4456	0.3756	0.0845	0.101*	
C16	0.2935 (9)	0.3901 (3)	0.0789 (8)	0.083 (3)	
H16	0.2636	0.3595	0.0546	0.100*	
C17	0.2244 (8)	0.4257 (3)	0.0947 (7)	0.073 (2)	
H17	0.1492	0.4196	0.0792	0.087*	
C18	0.2741 (7)	0.4699 (3)	0.1344 (6)	0.060 (2)	
H18	0.2313	0.4938	0.1475	0.072*	
C19	0.5921 (6)	0.5342 (3)	0.2423 (5)	0.0485 (17)	
C20	0.6401 (7)	0.5499 (3)	0.1748 (6)	0.060 (2)	
H20	0.5952	0.5659	0.1120	0.072*	
C21	0.7526 (7)	0.5422 (3)	0.1992 (7)	0.068 (2)	
H21	0.7828	0.5533	0.1536	0.081*	
C22	0.8193 (7)	0.5184 (3)	0.2904 (7)	0.064 (2)	
H22	0.8946	0.5123	0.3060	0.077*	
C23	0.7752 (7)	0.5031 (3)	0.3599 (7)	0.074 (2)	
H23	0.8209	0.4876	0.4231	0.089*	
C24	0.6624 (7)	0.5111 (3)	0.3344 (6)	0.063 (2)	
H24	0.6331	0.5006	0.3812	0.076*	
C25	0.1225 (6)	0.5213 (3)	0.2757 (6)	0.0512 (18)	
C26	0.1505 (7)	0.4826 (3)	0.3480 (6)	0.061 (2)	
H26	0.2064	0.4864	0.4167	0.073*	
C27	0.0942 (7)	0.4386 (3)	0.3166 (8)	0.071 (2)	
H27	0.1140	0.4128	0.3641	0.085*	
C28	0.0077 (7)	0.4326 (4)	0.2137 (8)	0.077 (3)	
H28	−0.0296	0.4030	0.1927	0.093*	
C29	−0.0202 (7)	0.4710 (3)	0.1457 (8)	0.072 (2)	
H29	−0.0781	0.4678	0.0779	0.086*	
C30	0.0366 (6)	0.5151 (3)	0.1763 (6)	0.0582 (19)	
H30	0.0160	0.5409	0.1284	0.070*	
C31	0.1930 (6)	0.5960 (3)	0.4352 (6)	0.0567 (19)	
C32	0.0874 (7)	0.5932 (3)	0.4398 (7)	0.070 (2)	
H32	0.0263	0.5787	0.3847	0.084*	
C33	0.0749 (9)	0.6121 (4)	0.5264 (7)	0.079 (3)	
H33	0.0043	0.6124	0.5273	0.094*	
C34	0.1650 (8)	0.6302 (4)	0.6095 (7)	0.080 (3)	
H34	0.1563	0.6413	0.6688	0.096*	
C35	0.2679 (9)	0.6326 (4)	0.6087 (7)	0.089 (3)	
H35	0.3288	0.6454	0.6667	0.107*	
C36	0.2826 (7)	0.6156 (3)	0.5201 (6)	0.070 (2)	
H36	0.3529	0.6177	0.5189	0.083*	
C37	0.1303 (6)	0.6189 (3)	0.2139 (6)	0.0516 (18)	
C38	0.0403 (8)	0.6452 (3)	0.2120 (7)	0.079 (3)	
H38	0.0229	0.6437	0.2702	0.095*	
C39	−0.0260 (8)	0.6740 (4)	0.1252 (8)	0.091 (3)	
H39	−0.0890	0.6901	0.1241	0.109*	
C40	0.0028 (9)	0.6785 (3)	0.0414 (7)	0.082 (3)	
H40	−0.0394	0.6984	−0.0158	0.098*	
C41	0.0906 (9)	0.6544 (4)	0.0423 (7)	0.086 (3)	
H41	0.1109	0.6574	−0.0139	0.104*	
C42	0.1533 (8)	0.6240 (3)	0.1295 (7)	0.075 (3)	
H42	0.2137	0.6067	0.1281	0.090*	
C43	0.709 (2)	0.2027 (8)	0.109 (2)	0.131 (7)	0.513 (4)
H43A	0.7768	0.2211	0.1497	0.157*	0.513 (4)
H43B	0.6878	0.2098	0.0346	0.157*	0.513 (4)
C44	0.325 (7)	0.255 (3)	0.169 (7)	0.131 (7)	0.173 (5)
H44A	0.3341	0.2214	0.1924	0.157*	0.173 (5)
H44B	0.2618	0.2690	0.1780	0.157*	0.173 (5)
C45	0.798 (7)	0.204 (3)	0.328 (8)	0.131 (7)	0.141 (5)
H45A	0.7746	0.1705	0.3323	0.157*	0.141 (5)
H45B	0.8602	0.2021	0.3081	0.157*	0.141 (5)
C46	0.330 (9)	0.260 (4)	0.020 (11)	0.131 (7)	0.173 (5)
H46A	0.3051	0.2395	−0.0415	0.157*	0.173 (5)
H46B	0.3070	0.2935	−0.0035	0.157*	0.173 (5)

### Atomic displacement parameters (Å^2^)

**Table:**

	*U*^11^	*U*^22^	*U*^33^	*U*^12^	*U*^13^	*U*^23^
Cu1	0.0449 (5)	0.0480 (5)	0.0388 (5)	0.0011 (4)	0.0178 (4)	−0.0006 (4)
F1	0.133 (10)	0.127 (17)	0.133 (11)	0.000 (9)	0.059 (8)	0.021 (10)
F2	0.16 (2)	0.080 (8)	0.176 (13)	0.035 (10)	0.036 (12)	0.016 (7)
F3	0.15 (3)	0.182 (16)	0.139 (15)	0.01 (2)	0.07 (2)	−0.059 (11)
F4	0.095 (10)	0.135 (18)	0.157 (12)	−0.007 (13)	−0.014 (8)	−0.005 (15)
F1'	0.16 (5)	0.14 (3)	0.18 (3)	−0.01 (3)	0.03 (3)	0.03 (2)
F2'	0.12 (3)	0.12 (3)	0.16 (3)	0.02 (3)	0.02 (2)	0.01 (3)
F3'	0.13 (2)	0.13 (4)	0.13 (2)	0.00 (2)	0.059 (16)	−0.01 (2)
F4'	0.10 (3)	0.13 (3)	0.14 (3)	0.00 (3)	0.05 (3)	−0.039 (19)
N1	0.052 (4)	0.038 (3)	0.050 (3)	0.001 (3)	0.021 (3)	0.000 (3)
N2	0.047 (3)	0.044 (3)	0.044 (3)	0.006 (3)	0.023 (3)	0.000 (3)
Cl1	0.115 (4)	0.079 (3)	0.200 (6)	0.004 (3)	0.042 (4)	0.054 (4)
Cl2	0.141 (6)	0.136 (6)	0.219 (8)	0.004 (5)	0.083 (6)	0.030 (5)
Cl3	0.115 (4)	0.079 (3)	0.200 (6)	0.004 (3)	0.042 (4)	0.054 (4)
Cl4	0.141 (6)	0.136 (6)	0.219 (8)	0.004 (5)	0.083 (6)	0.030 (5)
Cl5	0.115 (4)	0.079 (3)	0.200 (6)	0.004 (3)	0.042 (4)	0.054 (4)
Cl6	0.141 (6)	0.136 (6)	0.219 (8)	0.004 (5)	0.083 (6)	0.030 (5)
Cl7	0.115 (4)	0.079 (3)	0.200 (6)	0.004 (3)	0.042 (4)	0.054 (4)
Cl8	0.141 (6)	0.136 (6)	0.219 (8)	0.004 (5)	0.083 (6)	0.030 (5)
P1	0.0456 (11)	0.0486 (11)	0.0392 (9)	0.0020 (8)	0.0200 (8)	0.0000 (8)
P2	0.0431 (10)	0.0539 (11)	0.0409 (10)	0.0029 (8)	0.0190 (8)	0.0010 (8)
B1	0.124 (13)	0.080 (10)	0.093 (10)	0.018 (10)	0.033 (10)	−0.015 (8)
C1	0.068 (6)	0.052 (5)	0.066 (5)	0.001 (4)	0.022 (4)	−0.005 (4)
C2	0.082 (7)	0.052 (5)	0.092 (7)	−0.011 (5)	0.019 (5)	0.001 (5)
C3	0.081 (7)	0.064 (6)	0.091 (7)	−0.014 (5)	0.009 (5)	−0.014 (5)
C4	0.067 (6)	0.058 (5)	0.071 (5)	−0.004 (4)	0.005 (4)	−0.007 (4)
C5	0.049 (4)	0.049 (4)	0.056 (4)	0.003 (3)	0.022 (4)	−0.002 (3)
C6	0.050 (4)	0.051 (4)	0.042 (4)	0.005 (3)	0.018 (3)	0.000 (3)
C7	0.049 (4)	0.056 (5)	0.046 (4)	0.005 (3)	0.025 (3)	0.002 (3)
C8	0.085 (6)	0.062 (5)	0.050 (4)	−0.004 (5)	0.025 (4)	0.001 (4)
C9	0.101 (8)	0.062 (6)	0.072 (6)	0.000 (5)	0.029 (5)	0.006 (5)
C10	0.088 (7)	0.075 (6)	0.061 (5)	−0.002 (5)	0.030 (5)	0.017 (5)
C11	0.076 (6)	0.074 (6)	0.052 (5)	−0.001 (5)	0.028 (4)	0.001 (4)
C12	0.058 (5)	0.054 (4)	0.047 (4)	0.006 (4)	0.021 (3)	−0.001 (3)
C13	0.052 (4)	0.047 (4)	0.050 (4)	0.003 (3)	0.030 (3)	0.001 (3)
C14	0.063 (5)	0.057 (5)	0.082 (6)	−0.005 (4)	0.036 (5)	−0.010 (4)
C15	0.083 (7)	0.066 (6)	0.107 (8)	0.002 (5)	0.043 (6)	−0.021 (5)
C16	0.081 (7)	0.065 (6)	0.104 (7)	−0.019 (5)	0.039 (6)	−0.016 (5)
C17	0.070 (6)	0.074 (6)	0.075 (6)	−0.016 (5)	0.032 (5)	−0.016 (5)
C18	0.063 (5)	0.060 (5)	0.062 (5)	0.005 (4)	0.032 (4)	−0.007 (4)
C19	0.051 (4)	0.055 (4)	0.043 (4)	0.000 (3)	0.023 (3)	−0.001 (3)
C20	0.052 (5)	0.072 (5)	0.057 (5)	0.003 (4)	0.024 (4)	0.007 (4)
C21	0.056 (5)	0.086 (6)	0.073 (6)	−0.009 (5)	0.038 (5)	−0.003 (5)
C22	0.050 (5)	0.074 (6)	0.077 (6)	0.003 (4)	0.035 (4)	−0.007 (5)
C23	0.052 (5)	0.095 (7)	0.073 (6)	0.021 (5)	0.023 (4)	0.009 (5)
C24	0.057 (5)	0.077 (6)	0.063 (5)	0.011 (4)	0.033 (4)	0.009 (4)
C25	0.049 (4)	0.055 (4)	0.057 (4)	0.004 (4)	0.029 (4)	0.003 (3)
C26	0.062 (5)	0.061 (5)	0.064 (5)	0.004 (4)	0.031 (4)	0.006 (4)
C27	0.071 (6)	0.066 (6)	0.090 (6)	0.003 (5)	0.048 (5)	0.013 (5)
C28	0.061 (6)	0.074 (6)	0.099 (7)	−0.006 (5)	0.036 (5)	−0.003 (5)
C29	0.060 (6)	0.077 (6)	0.077 (6)	−0.011 (5)	0.028 (4)	−0.009 (5)
C30	0.055 (5)	0.062 (5)	0.059 (5)	−0.005 (4)	0.026 (4)	0.002 (4)
C31	0.054 (5)	0.071 (5)	0.049 (4)	0.011 (4)	0.026 (4)	0.003 (4)
C32	0.068 (6)	0.087 (6)	0.059 (5)	0.005 (5)	0.030 (4)	0.001 (4)
C33	0.080 (7)	0.106 (7)	0.070 (6)	0.016 (6)	0.051 (5)	0.005 (5)
C34	0.079 (7)	0.114 (8)	0.059 (5)	0.015 (6)	0.041 (5)	−0.005 (5)
C35	0.085 (7)	0.115 (8)	0.058 (5)	0.007 (6)	0.022 (5)	−0.021 (5)
C36	0.061 (5)	0.094 (7)	0.055 (5)	0.000 (5)	0.025 (4)	−0.010 (4)
C37	0.052 (5)	0.059 (5)	0.052 (4)	0.004 (4)	0.030 (4)	0.003 (3)
C38	0.080 (6)	0.095 (7)	0.067 (6)	0.025 (6)	0.037 (5)	0.022 (5)
C39	0.085 (7)	0.101 (8)	0.085 (7)	0.047 (6)	0.035 (6)	0.026 (6)
C40	0.083 (7)	0.081 (7)	0.070 (6)	0.017 (5)	0.021 (5)	0.023 (5)
C41	0.095 (7)	0.102 (8)	0.070 (6)	0.026 (6)	0.042 (5)	0.032 (5)
C42	0.075 (6)	0.089 (7)	0.068 (5)	0.025 (5)	0.037 (5)	0.019 (5)
C43	0.114 (17)	0.079 (13)	0.18 (2)	−0.002 (11)	0.048 (14)	0.025 (14)
C44	0.114 (17)	0.079 (13)	0.18 (2)	−0.002 (11)	0.048 (14)	0.025 (14)
C45	0.114 (17)	0.079 (13)	0.18 (2)	−0.002 (11)	0.048 (14)	0.025 (14)
C46	0.114 (17)	0.079 (13)	0.18 (2)	−0.002 (11)	0.048 (14)	0.025 (14)

### Geometric parameters (Å, °)

**Table:**

Cu1—N1	2.061 (6)	C16—C17	1.412 (12)
Cu1—N2	2.175 (5)	C16—H16	0.9300
Cu1—P1	2.2741 (19)	C17—C18	1.379 (11)
Cu1—P2	2.292 (2)	C17—H17	0.9300
F1—B1	1.43 (2)	C18—H18	0.9300
F2—B1	1.35 (2)	C19—C24	1.374 (10)
F3—B1	1.36 (4)	C19—C20	1.402 (10)
F4—B1	1.33 (3)	C20—C21	1.379 (11)
F1'—B1	1.40 (4)	C20—H20	0.9300
F2'—B1	1.39 (5)	C21—C22	1.364 (12)
F3'—B1	1.36 (3)	C21—H21	0.9300
F4'—B1	1.36 (9)	C22—C23	1.385 (12)
N1—C1	1.338 (9)	C22—H22	0.9300
N1—C5	1.361 (9)	C23—C24	1.382 (11)
N2—C6	1.278 (8)	C23—H23	0.9300
N2—N2^i^	1.428 (10)	C24—H24	0.9300
Cl1—C43	1.72 (2)	C25—C30	1.373 (10)
Cl2—C43	1.66 (3)	C25—C26	1.402 (10)
Cl3—C44	1.75 (8)	C26—C27	1.386 (11)
Cl4—C44	1.72 (9)	C26—H26	0.9300
Cl5—C45	1.74 (9)	C27—C28	1.407 (13)
Cl6—C45	1.69 (10)	C27—H27	0.9300
Cl7—C46	1.75 (12)	C28—C29	1.361 (12)
Cl8—C46	1.71 (11)	C28—H28	0.9300
P1—C13	1.820 (7)	C29—C30	1.391 (11)
P1—C7	1.823 (7)	C29—H29	0.9300
P1—C19	1.825 (7)	C30—H30	0.9300
P2—C37	1.819 (7)	C31—C36	1.373 (11)
P2—C31	1.833 (7)	C31—C32	1.414 (11)
P2—C25	1.833 (8)	C32—C33	1.384 (11)
C1—C2	1.432 (11)	C32—H32	0.9300
C1—H1	0.9300	C33—C34	1.347 (13)
C2—C3	1.346 (12)	C33—H33	0.9300
C2—H2	0.9300	C34—C35	1.354 (13)
C3—C4	1.378 (12)	C34—H34	0.9300
C3—H3	0.9300	C35—C36	1.404 (11)
C4—C5	1.391 (10)	C35—H35	0.9300
C4—H4	0.9300	C36—H36	0.9300
C5—C6	1.444 (10)	C37—C42	1.338 (11)
C6—H6	0.9300	C37—C38	1.372 (11)
C7—C12	1.374 (9)	C38—C39	1.396 (12)
C7—C8	1.381 (10)	C38—H38	0.9300
C8—C9	1.380 (11)	C39—C40	1.374 (13)
C8—H8	0.9300	C39—H39	0.9300
C9—C10	1.374 (12)	C40—C41	1.322 (13)
C9—H9	0.9300	C40—H40	0.9300
C10—C11	1.358 (11)	C41—C42	1.413 (11)
C10—H10	0.9300	C41—H41	0.9300
C11—C12	1.386 (10)	C42—H42	0.9300
C11—H11	0.9300	C43—H43A	0.9700
C12—H12	0.9300	C43—H43B	0.9700
C13—C14	1.365 (10)	C44—H44A	0.9700
C13—C18	1.408 (10)	C44—H44B	0.9700
C14—C15	1.412 (11)	C45—H45A	0.9700
C14—H14	0.9300	C45—H45B	0.9700
C15—C16	1.364 (13)	C46—H46A	0.9700
C15—H15	0.9300	C46—H46B	0.9700
			
N1—Cu1—N2	77.5 (2)	C21—C20—C19	121.6 (7)
N1—Cu1—P1	111.87 (17)	C21—C20—H20	119.2
N2—Cu1—P1	108.09 (15)	C19—C20—H20	119.2
N1—Cu1—P2	112.10 (17)	C22—C21—C20	119.9 (8)
N2—Cu1—P2	113.75 (15)	C22—C21—H21	120.1
P1—Cu1—P2	124.02 (7)	C20—C21—H21	120.1
C1—N1—C5	116.9 (6)	C21—C22—C23	120.1 (8)
C1—N1—Cu1	128.0 (5)	C21—C22—H22	119.9
C5—N1—Cu1	115.2 (5)	C23—C22—H22	119.9
C6—N2—N2^i^	113.5 (7)	C24—C23—C22	119.3 (8)
C6—N2—Cu1	112.5 (4)	C24—C23—H23	120.4
N2^i^—N2—Cu1	133.9 (6)	C22—C23—H23	120.4
C13—P1—C7	103.8 (3)	C19—C24—C23	122.2 (8)
C13—P1—C19	102.7 (3)	C19—C24—H24	118.9
C7—P1—C19	103.0 (3)	C23—C24—H24	118.9
C13—P1—Cu1	113.3 (2)	C30—C25—C26	118.7 (7)
C7—P1—Cu1	114.9 (2)	C30—C25—P2	124.0 (6)
C19—P1—Cu1	117.4 (2)	C26—C25—P2	117.1 (6)
C37—P2—C31	104.9 (3)	C27—C26—C25	119.6 (8)
C37—P2—C25	101.8 (3)	C27—C26—H26	120.2
C31—P2—C25	103.1 (3)	C25—C26—H26	120.2
C37—P2—Cu1	114.8 (2)	C26—C27—C28	121.0 (8)
C31—P2—Cu1	116.1 (3)	C26—C27—H27	119.5
C25—P2—Cu1	114.5 (2)	C28—C27—H27	119.5
F4—B1—F2	114.7 (17)	C29—C28—C27	118.5 (9)
F4'—B1—F3'	113 (3)	C29—C28—H28	120.8
F4—B1—F3	111 (2)	C27—C28—H28	120.8
F2—B1—F3	112 (2)	C28—C29—C30	120.9 (9)
F4'—B1—F2'	109 (4)	C28—C29—H29	119.5
F3'—B1—F2'	111 (4)	C30—C29—H29	119.5
F4'—B1—F1'	110 (4)	C25—C30—C29	121.3 (8)
F3'—B1—F1'	110 (2)	C25—C30—H30	119.4
F2'—B1—F1'	104 (3)	C29—C30—H30	119.4
F4—B1—F1	108.5 (19)	C36—C31—C32	118.5 (7)
F2—B1—F1	105.0 (17)	C36—C31—P2	119.9 (6)
F3—B1—F1	105 (2)	C32—C31—P2	121.5 (6)
N1—C1—C2	122.5 (8)	C33—C32—C31	119.9 (8)
N1—C1—H1	118.7	C33—C32—H32	120.1
C2—C1—H1	118.7	C31—C32—H32	120.1
C3—C2—C1	119.2 (8)	C34—C33—C32	120.0 (9)
C3—C2—H2	120.4	C34—C33—H33	120.0
C1—C2—H2	120.4	C32—C33—H33	120.0
C2—C3—C4	118.8 (8)	C33—C34—C35	121.6 (8)
C2—C3—H3	120.6	C33—C34—H34	119.2
C4—C3—H3	120.6	C35—C34—H34	119.2
C3—C4—C5	120.1 (8)	C34—C35—C36	119.9 (9)
C3—C4—H4	120.0	C34—C35—H35	120.1
C5—C4—H4	120.0	C36—C35—H35	120.1
N1—C5—C4	122.4 (7)	C31—C36—C35	120.0 (8)
N1—C5—C6	115.5 (6)	C31—C36—H36	120.0
C4—C5—C6	122.1 (7)	C35—C36—H36	120.0
N2—C6—C5	119.1 (6)	C42—C37—C38	116.0 (7)
N2—C6—H6	120.4	C42—C37—P2	119.4 (6)
C5—C6—H6	120.4	C38—C37—P2	124.4 (6)
C12—C7—C8	118.4 (7)	C37—C38—C39	122.0 (8)
C12—C7—P1	123.6 (6)	C37—C38—H38	119.0
C8—C7—P1	118.0 (5)	C39—C38—H38	119.0
C9—C8—C7	121.6 (8)	C40—C39—C38	119.3 (9)
C9—C8—H8	119.2	C40—C39—H39	120.4
C7—C8—H8	119.2	C38—C39—H39	120.4
C10—C9—C8	118.8 (8)	C41—C40—C39	119.9 (8)
C10—C9—H9	120.6	C41—C40—H40	120.1
C8—C9—H9	120.6	C39—C40—H40	120.1
C11—C10—C9	120.3 (8)	C40—C41—C42	119.4 (9)
C11—C10—H10	119.8	C40—C41—H41	120.3
C9—C10—H10	119.8	C42—C41—H41	120.3
C10—C11—C12	120.7 (8)	C37—C42—C41	123.3 (8)
C10—C11—H11	119.6	C37—C42—H42	118.4
C12—C11—H11	119.6	C41—C42—H42	118.4
C7—C12—C11	120.0 (7)	Cl2—C43—Cl1	119.5 (15)
C7—C12—H12	120.0	Cl2—C43—H43A	107.4
C11—C12—H12	120.0	Cl1—C43—H43A	107.4
C14—C13—C18	119.0 (7)	Cl2—C43—H43B	107.4
C14—C13—P1	123.1 (6)	Cl1—C43—H43B	107.4
C18—C13—P1	117.8 (5)	H43A—C43—H43B	107.0
C13—C14—C15	120.0 (8)	Cl4—C44—Cl3	106 (5)
C13—C14—H14	120.0	Cl4—C44—H44A	110.5
C15—C14—H14	120.0	Cl3—C44—H44A	110.5
C16—C15—C14	119.8 (9)	Cl4—C44—H44B	110.5
C16—C15—H15	120.1	Cl3—C44—H44B	110.5
C14—C15—H15	120.1	H44A—C44—H44B	108.7
C15—C16—C17	121.9 (9)	Cl6—C45—Cl5	116 (5)
C15—C16—H16	119.0	Cl6—C45—H45A	108.4
C17—C16—H16	119.0	Cl5—C45—H45A	108.4
C18—C17—C16	116.7 (8)	Cl6—C45—H45B	108.4
C18—C17—H17	121.7	Cl5—C45—H45B	108.4
C16—C17—H17	121.7	H45A—C45—H45B	107.4
C17—C18—C13	122.6 (7)	Cl8—C46—Cl7	107 (7)
C17—C18—H18	118.7	Cl8—C46—H46A	110.2
C13—C18—H18	118.7	Cl7—C46—H46A	110.2
C24—C19—C20	116.9 (7)	Cl8—C46—H46B	110.2
C24—C19—P1	120.1 (5)	Cl7—C46—H46B	109.0
C20—C19—P1	123.0 (6)	H46A—C46—H46B	108.5
			
N2—Cu1—N1—C1	179.0 (7)	C18—C13—C14—C15	1.0 (12)
P1—Cu1—N1—C1	74.3 (6)	P1—C13—C14—C15	179.4 (7)
P2—Cu1—N1—C1	−70.2 (7)	C13—C14—C15—C16	−1.7 (14)
N2—Cu1—N1—C5	−2.5 (5)	C14—C15—C16—C17	2.3 (16)
P1—Cu1—N1—C5	−107.3 (5)	C15—C16—C17—C18	−2.3 (15)
P2—Cu1—N1—C5	108.3 (5)	C16—C17—C18—C13	1.6 (13)
N1—Cu1—N2—C6	2.1 (5)	C14—C13—C18—C17	−1.1 (12)
P1—Cu1—N2—C6	111.4 (5)	P1—C13—C18—C17	−179.6 (6)
P2—Cu1—N2—C6	−106.8 (5)	C13—P1—C19—C24	−79.3 (7)
N1—Cu1—N2—N2^i^	−178.3 (8)	C7—P1—C19—C24	173.0 (6)
P1—Cu1—N2—N2^i^	−69.1 (7)	Cu1—P1—C19—C24	45.7 (7)
P2—Cu1—N2—N2^i^	72.8 (7)	C13—P1—C19—C20	96.9 (7)
N1—Cu1—P1—C13	159.0 (3)	C7—P1—C19—C20	−10.7 (7)
N2—Cu1—P1—C13	75.5 (3)	Cu1—P1—C19—C20	−138.0 (6)
P2—Cu1—P1—C13	−61.5 (3)	C24—C19—C20—C21	0.5 (12)
N1—Cu1—P1—C7	−81.9 (3)	P1—C19—C20—C21	−175.9 (6)
N2—Cu1—P1—C7	−165.4 (3)	C19—C20—C21—C22	0.7 (13)
P2—Cu1—P1—C7	57.6 (3)	C20—C21—C22—C23	−1.8 (13)
N1—Cu1—P1—C19	39.4 (3)	C21—C22—C23—C24	1.7 (13)
N2—Cu1—P1—C19	−44.0 (3)	C20—C19—C24—C23	−0.6 (12)
P2—Cu1—P1—C19	178.9 (3)	P1—C19—C24—C23	175.9 (7)
N1—Cu1—P2—C37	82.5 (3)	C22—C23—C24—C19	−0.5 (13)
N2—Cu1—P2—C37	168.2 (3)	C37—P2—C25—C30	15.0 (7)
P1—Cu1—P2—C37	−56.9 (3)	C31—P2—C25—C30	123.6 (7)
N1—Cu1—P2—C31	−40.3 (3)	Cu1—P2—C25—C30	−109.4 (6)
N2—Cu1—P2—C31	45.4 (3)	C37—P2—C25—C26	−169.6 (6)
P1—Cu1—P2—C31	−179.7 (3)	C31—P2—C25—C26	−61.0 (6)
N1—Cu1—P2—C25	−160.3 (3)	Cu1—P2—C25—C26	66.0 (6)
N2—Cu1—P2—C25	−74.7 (3)	C30—C25—C26—C27	2.5 (11)
P1—Cu1—P2—C25	60.3 (3)	P2—C25—C26—C27	−173.1 (6)
C5—N1—C1—C2	1.4 (12)	C25—C26—C27—C28	−1.4 (12)
Cu1—N1—C1—C2	179.8 (7)	C26—C27—C28—C29	−0.4 (13)
N1—C1—C2—C3	−3.8 (15)	C27—C28—C29—C30	1.1 (13)
C1—C2—C3—C4	4.7 (16)	C26—C25—C30—C29	−1.9 (11)
C2—C3—C4—C5	−3.4 (16)	P2—C25—C30—C29	173.4 (6)
C1—N1—C5—C4	−0.1 (11)	C28—C29—C30—C25	0.1 (13)
Cu1—N1—C5—C4	−178.7 (6)	C37—P2—C31—C36	−110.6 (7)
C1—N1—C5—C6	−178.7 (7)	C25—P2—C31—C36	143.2 (7)
Cu1—N1—C5—C6	2.6 (8)	Cu1—P2—C31—C36	17.3 (8)
C3—C4—C5—N1	1.1 (14)	C37—P2—C31—C32	67.5 (7)
C3—C4—C5—C6	179.6 (8)	C25—P2—C31—C32	−38.7 (7)
N2^i^—N2—C6—C5	178.9 (6)	Cu1—P2—C31—C32	−164.6 (6)
Cu1—N2—C6—C5	−1.4 (8)	C36—C31—C32—C33	3.0 (13)
N1—C5—C6—N2	−0.7 (10)	P2—C31—C32—C33	−175.1 (7)
C4—C5—C6—N2	−179.4 (7)	C31—C32—C33—C34	−4.6 (14)
C13—P1—C7—C12	−6.9 (7)	C32—C33—C34—C35	3.3 (16)
C19—P1—C7—C12	99.9 (7)	C33—C34—C35—C36	−0.5 (16)
Cu1—P1—C7—C12	−131.2 (6)	C32—C31—C36—C35	−0.2 (13)
C13—P1—C7—C8	175.2 (6)	P2—C31—C36—C35	177.9 (7)
C19—P1—C7—C8	−78.0 (7)	C34—C35—C36—C31	−1.1 (15)
Cu1—P1—C7—C8	50.9 (7)	C31—P2—C37—C42	159.1 (7)
C12—C7—C8—C9	3.5 (13)	C25—P2—C37—C42	−93.7 (7)
P1—C7—C8—C9	−178.4 (7)	Cu1—P2—C37—C42	30.5 (8)
C7—C8—C9—C10	−2.1 (15)	C31—P2—C37—C38	−25.2 (9)
C8—C9—C10—C11	−0.8 (15)	C25—P2—C37—C38	82.0 (8)
C9—C10—C11—C12	2.0 (14)	Cu1—P2—C37—C38	−153.8 (7)
C8—C7—C12—C11	−2.2 (11)	C42—C37—C38—C39	2.4 (14)
P1—C7—C12—C11	179.9 (6)	P2—C37—C38—C39	−173.3 (8)
C10—C11—C12—C7	−0.5 (13)	C37—C38—C39—C40	−3.5 (16)
C7—P1—C13—C14	100.6 (7)	C38—C39—C40—C41	2.0 (16)
C19—P1—C13—C14	−6.4 (7)	C39—C40—C41—C42	0.4 (16)
Cu1—P1—C13—C14	−134.1 (6)	C38—C37—C42—C41	0.1 (14)
C7—P1—C13—C18	−81.0 (6)	P2—C37—C42—C41	176.1 (8)
C19—P1—C13—C18	172.0 (6)	C40—C41—C42—C37	−1.6 (16)
Cu1—P1—C13—C18	44.3 (6)		

Symmetry codes: (i) −*x*+1, −*y*+1, −*z*+1.
